# Thyroid dysfunction in Khyber Pakhtunkhwa, Pakistan

**DOI:** 10.12669/pjms.321.8476

**Published:** 2016

**Authors:** Shahnaz Attaullah, Bibi Safia Haq, Mairman Muska

**Affiliations:** 1 Dr. Shahnaz Attaullah, Ph.D (Biochemistry), Assistant Professor, Department of Chemistry, Jinnah College for Women, University of Peshawar, Peshawar, Pakistan; 2 Dr. Bibi Safia Haq, Ph.D (Medical Physics), Assistant Professor, Department of Physics, Jinnah College for Women, University of Peshawar, Peshawar, Pakistan; 3 Mairman Muska, M. Phil, Lecturer, Department of Chemistry, Jinnah College for Women, University of Peshawar, Peshawar, Pakistan

**Keywords:** Tri-iodothyronine, Tetra-iodothyronine, Thyroidstimulating hormone, Thyroid dysfunction

## Abstract

**Objectives:**

The objective of this research was to elucidate some of the major relation of thyroid dysfunctions, keeping in view the various selected demographic details of included patients.

**Methods:**

This study was approved by the ethical committee of Post Graduate Medical Institute (PGMI) Hayatabad Medical Complex Peshawar, and was conducted in the Institute of Radioactive Nuclear Medicine (IRNUM) Peshawar. The blood samples were collected, followed by their analysis for triiodothyronine (T_3_), tetraiodothyronine (T_4_) and thyroid stimulating hormone (TSH).

**Results:**

The results obtained regarding the demographical aspects of the patients revealed that female gender has categorically significantly high percentage of occurrence of thyroid abnormality as compared to male gender (75.8% vs. 24.2%). Results regarding locality distribution of the patients depicted that majority of those belonged to the local population of Peshawar and Charsadda region.

**Conclusion:**

In Pakistan especially Khyber Pakhtunkwa (KPK), thyroid diseases are more common in females as compared to males. The most probable causes could be lactation and pregnancy.

## INTRODUCTION

Endocrine diseases are common, particularly those of the thyroid gland. Some endocrine glands respond directly to metabolic glands; while most are controlled by hormones released from the pituitary gland.^1^ Endocrine diseases are increasing globally but are growing more rapidly in Asia.^2^ In Nepal, about 0.2% of deaths are because of endocrine disorders, the major cause of which is iodine deficiency.^3^ The pathophysiology of many thyroid diseases relates to TSH, T_3_ and T_4_. The most important chemical marker of thyroid function is TSH. Hyperthyroidism is the result of low TSH profile, whereas high value leads to hypothyroidism.^4^

Thyrotoxicosis is the hyper-metabolic condition confirmed by estimation of free T4 (fT4), free T3 (fT3).^5^ Thyrotoxicosis or hyperthyroidism is an excess of thyroid hormone caused by an over active thyroid tissue or is the consequence of additional synthesis and liberation of thyroid hormone. The incidence of hyperthyroidism is lower i.e., 2% as compared with hypothyroidism in general population of Pakistan.^6^ In 90% cases, thyrotoxicosis or hyperthyroidism is caused by Graves’s disease, toxic multinodular goiter and toxic adenoma. In some cases, sub-acute thyroditis is also responsible for hyperthyroidism.^7^ After Graves’s disease, the major cause of thyrotoxicosis is toxic multinodular goiter, which has mostly been diagnosed in mature and aged patients. About 5% of patients with thyrotoxicosis have toxic thyroid adenoma caused by excessive release of thyroid hormones.^8^

Hypothyroidism is a common metabolic disorder in the general population. It is characterized by diminished metabolism, retarded growth and development, impaired mental activity and swelling of certain parts of the skin. It is a disease caused by lack of iodine in drinking water.^9^ Hypothyroidism is a clinical condition due to deficiency of thyroid hormone and increased level of TSH.^10^ Hypo-function of the thyroid gland is also accompanied by changes throughout the organism. Hypothyroidism was found in more than 2% of 2800 patients.^11^ In case of sufficient iodine intake; autoimmune thyroid disease Hashimoto’s thyroditis appears to be the most common cause. In this situation, there is replacement of normal thyroid tissue with lymphocytic and rubbery tissue.^12^ The presence of excess iodine in patient’s body is also a cause of hypothyroidism including patients with a history of radioactive therapy, autoimmune thyroditis and subtotal thyroidectomy. Other causes of hypothyroidism include certain medications like interferon alpha, amiodarone, thalidomide lithium and sta-vudine.^13^

## METHODS

Blood samples and data were collected from patients attending the Institute of Radioactive Nuclear Medicine (IRNUM) Peshawar, Pakistan. Patients belonging to different areas of Khyber Pakhtunkhwa were included in this study. The study subjects were divided into three groups: euthyroid, hypothyroid and hyperthyroid. Euthyriods serve as the control group; the number of cases included in this group is 214, whereas 195 and 191 patients are grouped as hyperthyroids and hypothyroids, respectively. Patients suffering from chronic diseases like hypertension, diabetes mellitus and cardiac disorders were excluded. Patients having thyroid surgery were also excluded. Patients who were taking thyroxin were also excluded. The selected cases were analyzed for serum levels of TSH, T_4_, T_3_ at IRNUM, Peshawar, Pakistan, by using Gamma counter. TSH in the sample is estimated by using radioimmunoassay (RIA).^14^ The principle involved in the determination of fT_4_ and fT_3_ is based on the use of labeled antibody.^15^,^16^

## RESULTS

**Fig.1:**
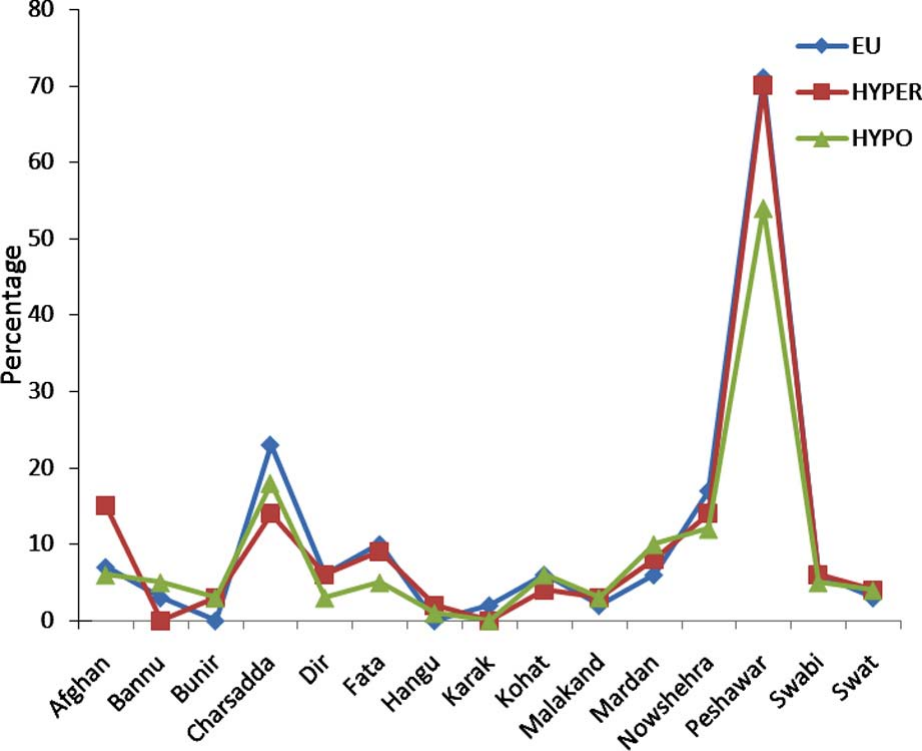
Comparison of Locality with clinical diagnosis (Female Population).

**Fig.2:**
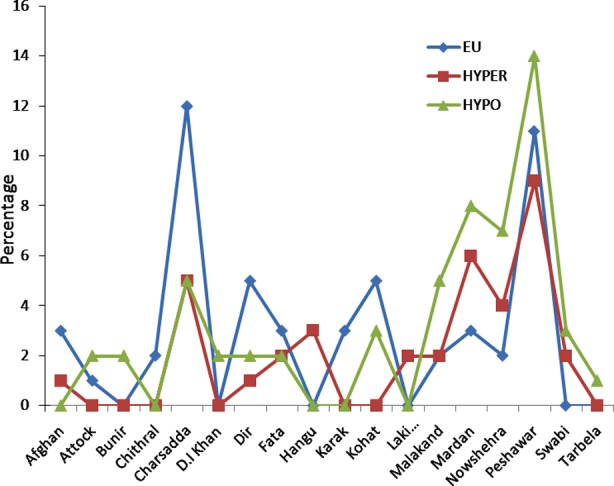
Comparison of Locality with clinical diagnosis (Male Population).

Results in Fig. 1 and 2 illustrate the area-wise distribution of both male and female patients and their clinical diagnosis. On the basis of thyroid function tests, a statistically significant variation in the mean levels of T_3_ was observed for hypothyroid (3.31 ± 0.128 pmol/L) and hyperthyroid (6.98 ± 0.367 pmol/L) when compared to the mean levels in controls i.e. euthyroid (3.92 ± 0.143 pmol/L). Similarly, significant differences were observed in the mean levels of T_4_ for hypothyroid (10.71 ± 0.637 pmol/L) and hyperthyroid (34.75 ± 1.640 pmol/L) when compared to the mean levels in controls i.e. euthyroid (17.10 ± 0.332 pmol/L). The study also showed significant changes in TSH i.e. in hypothyroid (31.47 ± 1.628 uIU/mL) and in hyperthyroid (0.25 ± 0.003 uIU/mL) compared to the mean in controls i.e. euthyroid (1.77 ± 0.130 uIU/mL).

**Table-I:** Comparison of descriptive of thyroid function tests with clinical diagnosis.

Laboratory Results N=600		EU (N=214)				Hyper (N=195)				Hypo(N=191)			

		Min	Max	Mean	SD	Min	Max	Mean	SD	Min	Max	Mean	SD
Thyroid Function Tests	T3 (pmol/L)	0.10	7.10	3.92	1.62	1.20	32.60	6.98	4.17	0.80	7.00	3.31	1.43
	T4 (pmol/L)	2.90	24.00	17.10	3.70	5.90	77.00	34.75	18.63	0.00	54.00	10.71	7.14
	TSH (uIU/ml)	0.20	10.40	1.77	1.44	0.00	0.13	0.25	0.36	0.06	51.00	31.47	18.27

### Statistical Analysis

Primary data ingress was processed on MS Database version 2007, which was cleaned in MS Excel with sophisticated formulas for corrections. Data were checked in EPI-info Version 16.0 for scientific verifications. The data were finally imported in SPSS version 20.0 for analysis. The student’s “t” test, is being used to find out the significance between two values, in various diseased groups. Frequencies, p-value, and other descriptive analysis was done to calculate the mean and standard deviation of different parameters.

The latest versions of the said programs were used for graphical and tabular analysis. The valid frequencies were premeditated for desired results. To compare the significance of the difference between the two means, their values are given as (Mean ± SD/SEM).

The results in Fig.1 show greater number of hyperthyroid and hypothyroid females from Peshawar city, followed by patients from Charsadda and those who migrated from Afghanistan.. However, there is a marked difference in the number of female subjects having thyroid dysfunction in Peshawar as compared to rest of the localities.

Just like the female population, increased numbers of thyroid disease cases in males were present in Peshawar region, with relatively greater number of hypothyroids (Fig.2). There were almost equal number of male patients suffering from hyperthyroidism and hypothyroidism belonging to Charsadda. The number of patients with thyroid dysfunction in rest of the areas in KPK as well as Afghanistan occurs in a similar patron.

**Table-II:** Association between descriptive of thyroid function tests with EU & Hyper.

Laboratory Results N=600		EU (N=214)				Hyper (N=195)				Association

		Min	Max	Mean	SD	Min	Max	Mean	SD	P-Value
Thyroid Function Tests	T3 (pmol/L)	0.10	7.10	3.92	1.62	1.20	32.60	6.98	4.17	0.999
	T4(pmol/L)	2.90	24.00	17.10	3.70	5.90	77.00	34.75	18.63	0.000
	TSH (uIU/ml)	0.20	10.40	1.77	1.44	0.00	0.13	0.25	0.36	0.264

**Table-III:** Association between descriptive of thyroid function tests with EU & Hypo.

Laboratory Results N=600		EU (N=214)				Hyper (N=191)				Association

		Min	Max	P-Value	SD	Min	Max	Mean	SD	P-Value
Thyroid Function Tests	T3 (pmol/L)	0.10	7.10	3.92	1.62	0.80	7.00	3.31	1.43	0.921
	T4 (pmol/L)	2.90	24.00	17.10	3.70	0.00	54.00	10.71	7.14	1.000
	TSH (uIU/ml)	0.20	10.40	1.77	1.44	0.06	51.00	31.47	18.27	0.666

## DISCUSSION

The pair sample “t” test showed that the mean difference of hyperthyroid patients with T_3_ is non-significant when compared with the control group. The difference was statistically significant for TSH and T_4_ in the same group. These findings are in accordance with the study of Tayal D et al.^17^

Thyroid dysfunction was diagnosed by the chemical analysis of thyroid function tests. The prevalence of diagnosis was 214 cases of Euthyroidism (35.7%), 195 Hyperthyroidism (32.5%) and 191 hypothyroidism (31.8%), respectively out of a total 600 cases. As regards the nature of abnormality of thyroid disorder, it has been observed that patients of Euthyroidismi-e., 214 were more than 191 and 195 for hyperthyroidism and hypothyroidism respectively. The prevalence of hypothyroidism is slightly higher than that of hyperthyroidism, as indicated in a study by Baral. The association of TFTs with hypothyroidism is statistically significant (p=0.005) for T_3_. The difference is highly significant for TSH and T_4_ as (p=0.000). Hypothyroidism is an observable fact. In the initial stage there is a minor decrease in T_4_ which leads to an elevated value of TSH whereas T_3_ are in the reference range. Due to the deficiency of thyroid hormone, a biochemical abnormality is present but is asymptomatic. Although in some severe cases the danger exists resulting in a life-threatening illness.^18^

The locality wise distribution and the frequencies of euthyroidism, hyperthyroidism and hypothyroidism of the selected cases was also noted: Patients from 20 different districts of KPK were included in the study. The majority of the patients were from the Peshawar and its nearby districts. A total of 229 (38.16%) subjects included 13.17% hyperthyroids and 11.3% hypothyroids. On the other hand 77 (12.83%) individuals were belonging to district Charsadda KPK, out of these 19(3.17%) turned out to be hyperthyroids and 23 (3.83%) as hypothyroids. A total of 56 (9.3%) patients belonged to district Nowshehra, out of these 18 (3%) were diagnosed as hyperthyroids and 19(3.17%) as hypothyroids. Similarly, a total of 41 (6.83%) patients were from the district Mardan, out of these 12 (2.33%) and 18 (3%) were hyperthyroids and hypothyroids, respectively. Patients from the nearby districts of Punjab i.e. Attock and Tarbela, and D.I. Khan from KPK were also included in the study. The prevalence of thyroid dysfunction is greater in Peshawar and in the three main districts i-e Charsadda, Nowshehra and Mardan.

According to the above results the incidence of hyperthyroidism in Peshawar is evident, whereas in the remaining three districts there is greater incidence of hypothyroidism. The reason is being greater population in Peshawar and availability of medical and laboratory facilities. Importantly, the data showed that significant percentage of patients were from Afghanistan. The higher occurrence of these disorders among foreign refugees may be because they are adversely affected by chronic exposure to the toxic radiations due to devastating war conditions, chemical weapons and bomb blasts from foreign invaders for the last three decades. It is also evident from the literature that 18% of the survivors of the atomic bomb explosion in Japan (Hiroshima Nagasaki) developed thyroid cancers especially those who are exposed to the carcinogenic effect of radiation during childhood.^19^

Beside these, the occurrences of thyroid dysfunctions in different regions were less than 5%. However, Hypothyroidism is more prevalent in Bunnu, Kohat, Malakand and Fata region. Cardiovascular diseases must be monitored especially angina, Ischemic heart disease as coronary atherosclerosis is common in hypothyroidism. Hypothyroidism can increase blood cholesterol levels and that contributes to heart disease; however, if the hypothyroidism is being treated with a thyroid hormone, then the cholesterol returns to normal.^20^ To prevent cardiovascular disease myxodema coma, hypothyroidism must be treated with thyroxin immediately and thyroid function should be monitored frequently with treatment. This study shows the frequency of the patients having problems of thyroid disorders in comparison with their locality. Results regarding locality distribution of the patients depicted that majority of these belong to local population of Peshawar and Charsadda region.

## CONCLUSION

In Pakistan especially KPK, thyroid diseases are more common in females as compared to males. The most probable cause could be lactation and pregnancy. In lactation and pregnancy BMR is raised because the body requirement increases than the normal, leading to stimulation of thyroid gland to produce more hormones. Early diagnosis and treatment should be done as early as possible. Another important conclusion is the commonness of thyroid disorders in Afghan refugees which is due to exposure to chemical weapons and radiations due to bomb blasts in Afghanistan and the nearby regions of KPK. As Peshawar is geographically close to Afghanistan so the people in KP are more prone to be affected by the radiations due to bomb blast. So nationally and internationally peace and harmony should be ensured, especially in KPK and Afghanistan.
